# Capsid display of a conserved human papillomavirus L2 peptide in the adenovirus 5 hexon protein: a candidate prophylactic hpv vaccine approach

**DOI:** 10.1186/s12985-015-0364-7

**Published:** 2015-09-11

**Authors:** Wai-Hong Wu, Tanwee Alkutkar, Balasubramanyan Karanam, Richard BS Roden, Gary Ketner, Okechukwu A. Ibeanu

**Affiliations:** Department of Pathology, Johns Hopkins University School of Medicine, Baltimore, MD USA; Department of Gynecology and Obstetrics, Johns Hopkins University School of Medicine, Baltimore, MD USA; Department of Oncology, Johns Hopkins University School of Medicine, Baltimore, MD USA; W. Harry Feinstone Department of Molecular Microbiology and Immunology, Johns Hopkins University Bloomberg School of Public Health, Baltimore, MD USA; Division of Gynecologic Oncology, Sinai Hospital of Baltimore, Baltimore, MD USA

## Abstract

**Background:**

Infection by any one of 15 high risk human papillomavirus (hrHPV) types causes most invasive cervical cancers. Their oncogenic genome is encapsidated by L1 (major) and L2 (minor) coat proteins. Current HPV prophylactic vaccines are composed of L1 virus-like particles (VLP) that elicit type restricted immunity. An N-terminal region of L2 protein identified by neutralizing monoclonal antibodies comprises a protective epitope conserved among HPV types, but it is weakly immunogenic compared to L1 VLP. The major antigenic capsid protein of adenovirus type 5 (Ad5) is hexon which contains 9 hypervariable regions (HVRs) that form the immunodominant neutralizing epitopes. Insertion of weakly antigenic foreign B cell epitopes into these HVRs has shown promise in eliciting robust neutralizing antibody responses. Thus here we sought to generate a broadly protective prophylactic HPV vaccine candidate by inserting a conserved protective L2 epitope into the Ad5 hexon protein for VLP-like display.

**Methods:**

Four recombinant adenoviruses were generated without significant compromise of viral replication by introduction of HPV16 amino acids L2 12–41 into Ad5 hexon, either by insertion into, or substitution of, either hexon HVR1 or HVR5.

**Results:**

Vaccination of mice three times with each of these L2-recombinant adenoviruses induced similarly robust adenovirus-specific serum antibody but weak titers against L2. These L2-specific responses were enhanced by vaccination in the presence of alum and monophoryl lipid A adjuvant. Sera obtained after the third immunization exhibited low neutralizing antibody titers against HPV16 and HPV73. L2-recombinant adenovirus vaccination without adjuvant provided partial protection of mice against HPV16 challenge to either the vagina or skin. In contrast, vaccination with each L2-recombinant adenovirus formulated in adjuvant provided robust protection against vaginal challenge with HPV16, but not against HPV56.

**Conclusion:**

We conclude that introduction of HPV16 L2 12–41 epitope into Ad5 hexon HVR1 or HVR5 is a feasible method of generating a protective HPV vaccine, but further optimization is required to strengthen the L2-specific response and broaden protection to the more diverse hrHPV.

## Introduction

Cervical cancer has an annual global incidence of approximately 500,000 cases, with about 250,000 disease related deaths. Most (>85 %) of these deaths occur in developing countries, notably in sub-Saharan Africa, southeast Asia, and South America, which currently harbor the heaviest disease burden [1]. It has been established that cervical cancer is mostly a sexually transmitted disease that results from persistent infection with 'high-risk' oncogenic human papillomaviruses (HPV), with such HPV DNA being present in >95 % of cervical cancers diagnosed [2]. The most prevalent oncogenic HPV types are 16 and 18 causing approximately 50 % and 20 % of cervical cancer cases respectively. A dozen other HPV types, notably HPV31, 33, 45, 52, 56, and 58 are also oncogenic, together accounting for about 30 % of cases, with each type responsible for a small fraction of cervical cancer [3]. An ideal HPV vaccine therefore would protect against all high risk HPV types [4].

Most HPV infections are asymptomatic or regress in immune-competent individuals, but it is the small fraction of persistent cases that can progress to cancer. Human papillomaviruses are non-enveloped, small DNA viruses with a genome that is approximately 8 kilobases and encodes eight genes; six early genes (*E1*, *E2*, *E4*, *E5*, *E6*, *E7*) and two late genes (*L1*, and *L2*) [5]. HPV infects basal cells in the epithelium following genital abrasions sustained during sexual activity with infected individuals. The early viral proteins affect the host cell cycle in favor of viral replication whereas the late proteins (L1 and L2) assemble into a capsid structure around the histone-bound viral genome to form new viral particles [6, 7]. Infectious viral particles are released during the natural process of shedding of the terminally differentiated epithelial cells, likely aided by E4’s capacity to disrupt keratin filaments [8]. HPV infection is highly prevalent among sexually active adults globally, and HPV-related diseases are expected to continue as a major global healthcare problem in developing countries due to limited implementation of accepted preventive measures.

The commercially available HPV vaccines are Gardasil (Merck and Company, NJ, USA), and Cervarix (GlaxoSmithKline, UK). Both vaccines are polyvalent, and contain viral-like particles (VLPs) assembled from the L1 major capsid protein of HPV 16 and 18 types. Gardasil also contains HPV 6 and 11 VLPs, and a new version, Gardasil 9, which also contains HPV31, 33, 45, 52, 58 L1 VLP was recently licensed [9]. While Gardasil 9 targets these dominant α7 and α9 species, it does not target all high risk HPV types, including HPV56 and HPV73 which are α6 and α11 species respectively. These vaccines do not contain live viral material, but induce both a potent neutralizing antibody response and durable protection against the targeted genotypes [10–13]. The immunodominant neutralizing epitopes in the hypervariable loops of the L1 protein are not cross-reactive among HPV types [14, 15]. Therefore, there is limited or ineffective protection against other oncogenic HPV types and highly multivalent formulations are required for broad coverage [11]. Other limitations of the current L1 based vaccines are cost, need for vaccine cold-chain, continued need for cervical screening, and the need for more than one injection to complete the vaccine course. Even in the United States, vaccination coverage remains low in adolescent females [16]. In many developing countries, national HPV vaccination programs do not even exist. There is a need for an alternative prophylactic HPV vaccine to expand access globally that is inexpensive, stable at tropical temperatures, broadly cross-protective against high risk HPV, and preferably administered as single dose for compliance [9].

The L2 protein is an immunogen whose N terminal region is highly conserved among HPV types and can elicit protective immunity in animal papillomavirus models [17]. L2 is required for infection, and its cleavage at residue 11 of HPV16 L2 by furin is an essential step [18]. Furthermore, a putative transmembrane domain between residues 45–67 of L2 is also critical, possibly to facilitate crossing the limiting membrane during infection [19]. Several groups have identified conserved protective epitopes between these regions, such as residues 17–36 [20] and 28–42 [21, 22] of HPV16 L2 [23], that can potentially serve as a single broadly protective immunogen. However, despite the use of strong adjuvants, these peptide epitopes are weakly immunogenic compared to L1 VLP [22].

A promising strategy to enhance the immune response to these L2-based protective peptides is their substitution for, or insertion into the immunodominant neutralizing epitope of a heterologous virus [24]. With the availability of detailed structural data for adenoviruses [25, 26], several investigators have used various adenovirus types as platforms for capsid display of foreign antigens to induce protective immunity against a host of human pathogens [27–29]. Such strategy exploits several potentially advantageous characteristics of human adenoviruses. Human adenoviruses typically cause non-fatal respiratory and gastrointestinal infections. Orally administered single dose vaccines containing lyophilized fully replication competent adenovirus 4 and 7 have been in use for decades, and have proven to be highly effective and safe [30, 31]. The hexon protein of adenovirus is exposed at the viral surface and contains nine hyper-variable regions (HVRs). The HVRs display adenoviral epitopes that are responsible for serotype-specific antigenicity, and their specific amino acid sequences can be freely modified [32]. The work that is presented in this paper is based on the hypothesis that it is feasible to generate protective neutralizing antibodies against a recombinant vaccine construct comprised of the HPV 16 L2 antigenic peptide sequence displayed in HVRs of the human Ad5 hexon capsid protein.

## Results

### Generation of recombinant adenoviruses displaying L2

Immunization of rabbits with a synthetic HPV16 L2 17–36 peptide coupled to KLH carrier or HPV16 L2 1–88 polypeptide in Freund’s adjuvant was previously shown to induce a moderate titer antibody response capable of neutralizing diverse HPV types [20, 23, 33]. To explore the potential of capsid display of L2 to trigger a robust cross-neutralizing antibody response without the use of adjuvant, we generated four recombinant adenoviruses. The HPV16 L2 12–41 region was selected for display since this spans the furin-cleavage site through to the edge of a putative transmembrane domain, and encompasses the RG-1 and other cross-protective epitopes at 17–36 [20] and 28–42 [21, 22]. The optimal approach to display the L2 epitope in the HVRs of hexon is unclear with respect to both viral propagation and immunogenicity. Therefore the L2 epitope was either inserted into the middle of an HVR, or used to replace the HVR sequence (Table 1). Likewise, adenovirus contains multiple HVR, and it is not known which is appropriate for display of L2. Since HVR1 and HVR5 have previously been used to display heterologous epitopes of 24 and 66 amino acids (reviewed in [34]), the L2 epitope was inserted or substituted into both (Table 1) by recombination. The presence of the desired HPV antigen in the hexon HVRs of plaque-purified recombinant adenoviruses was confirmed by Western blot using a monoclonal antibody (RG-1) against HPV16 L2 17–36 (prepared by Harlan Laboratories). Western blots revealed an RG1-reactive band of approximately 120 KDa in all four recombinant adenoviruses, consistent with the expected size of adenovirus hexon protein containing the L2 insert, whereas none reacted with negative control monoclonal antibody 2A10. Reaction with antibody to adenovirus capsid proteins by Western blot revealed a normal complement of adenovirus virion components. All four L2 recombinant adenoviruses replicated efficiently in 293 cells, as can be seen from the high titers observed for the standard virion preparations (Table 1).

**Table 1:** Summary of production of L2-recombinant Ad5 virions. Shown are the designation of each L2-recombinant adenovirus, the nature of the L2 insert, the site of hexon into which it was inserted, the mode of insertion and the pfu of virus obtained upon a standard preparation

Construct	Insert (a.a.)	Hexon site (a.a.)	Mode	Particles/ml
5 ins	HPV16	271–272	HVR5 Insertion	2.2 × 10^12^
	L2 12–41			
5 sub	HPV16	271–281	HVR5 Substitution	2.37 × 10^12^
	L2 12–41			
1 sub	HPV16	138–167	HVR1 Substitution	1.80 × 10^12^
	L2 12–41			
1 ins	HPV16	128–129	HVR1 Insertion	1.76 × 10^12^
	L2 12–41			

### Immunogenicity of L2-recombinant adenovirus

**Fig. 1:**
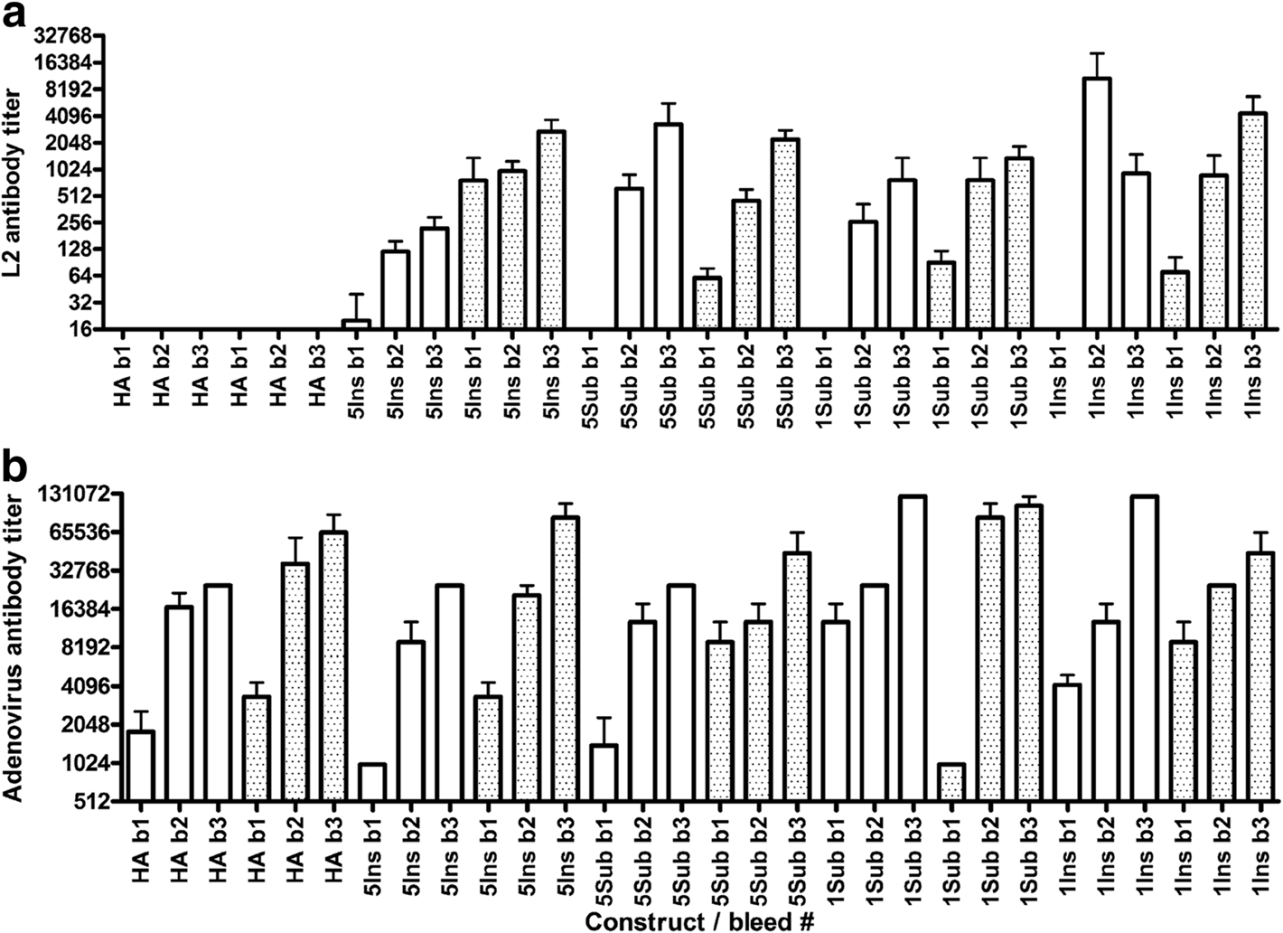
Adenovirus and L2-specific serum antibody response after vaccination of mice with L2-recombinant adenoviruses either with or without adjuvant. *Legend*: Balb/c mice (5/group) were vaccinated s.c. three times at 3 week intervals with 10^10^ L2 or HA-recombinant adenovirus particles either with 50 μg aluminum hydroxide gel and 5 μg monophosphoryl lipid A (shaded boxes) or without adjuvant (clear boxes) in a total volume of 150 μL/dose, or controls Gardasil (0.1x human dose) or buffer. Serum was collected 3 weeks after each immunization (i.e. bleed numbers b1, b2 or b3) and two-fold dilutions tested by ELISA. ELISA titers against adenovirus (**a**) or L2 (**b**) are shown as mean + SEM. HA: mice immunized with the HA recombinant control virus

Groups of 5 Balb/c mice were immunized three times at 3 week intervals either with recombinant adenoviruses displaying the L2 epitope or a recombinant that displays an irrelevant epitope. ELISA to detect antibodies against wild type Ad5 particles and HPV L2 were performed on the serum collected at 3 week intervals (Fig. 1a). Every mouse in each group produced antibodies against the adenovirus vector. In general, individual mice responded to the first immunization with anti-adenovirus titers of approximately 10^3^, with increases after each subsequent immunization to maximum titers >10^4^ after the third dose. The use of an adjuvant (alum and monophosphoryl lipid A) did boost the anti-adenovirus antibody titers. Overall, there was no clear difference in the induction of adenovirus particle-specific antibodies by the four L2 recombinant adenoviruses, suggesting that neither insertion into, nor substitution of HVR1 or HVR5 with the L2 peptide disrupts the capsid structure or antigenicity substantially.

Antibody titers to L2 were lower than to adenovirus, but increased after each boost (Fig. 1b). Furthermore, antibody responses to HPV L2 were noticeably increased with the use of adjuvant. There was no significant difference in the induction of L2-specific antibody by the four recombinant adenoviruses (Fig. 1b). Since Gardasil is made up of L1 viral like particles, the mice immunized with Gardasil produced no antibodies to HPV L2 or adenovirus, similar to the PBS negative control mice (not shown).

### Immunization with L2-recombinant adenoviruses induces antibodies that neutralize HPV

**Fig. 2:**
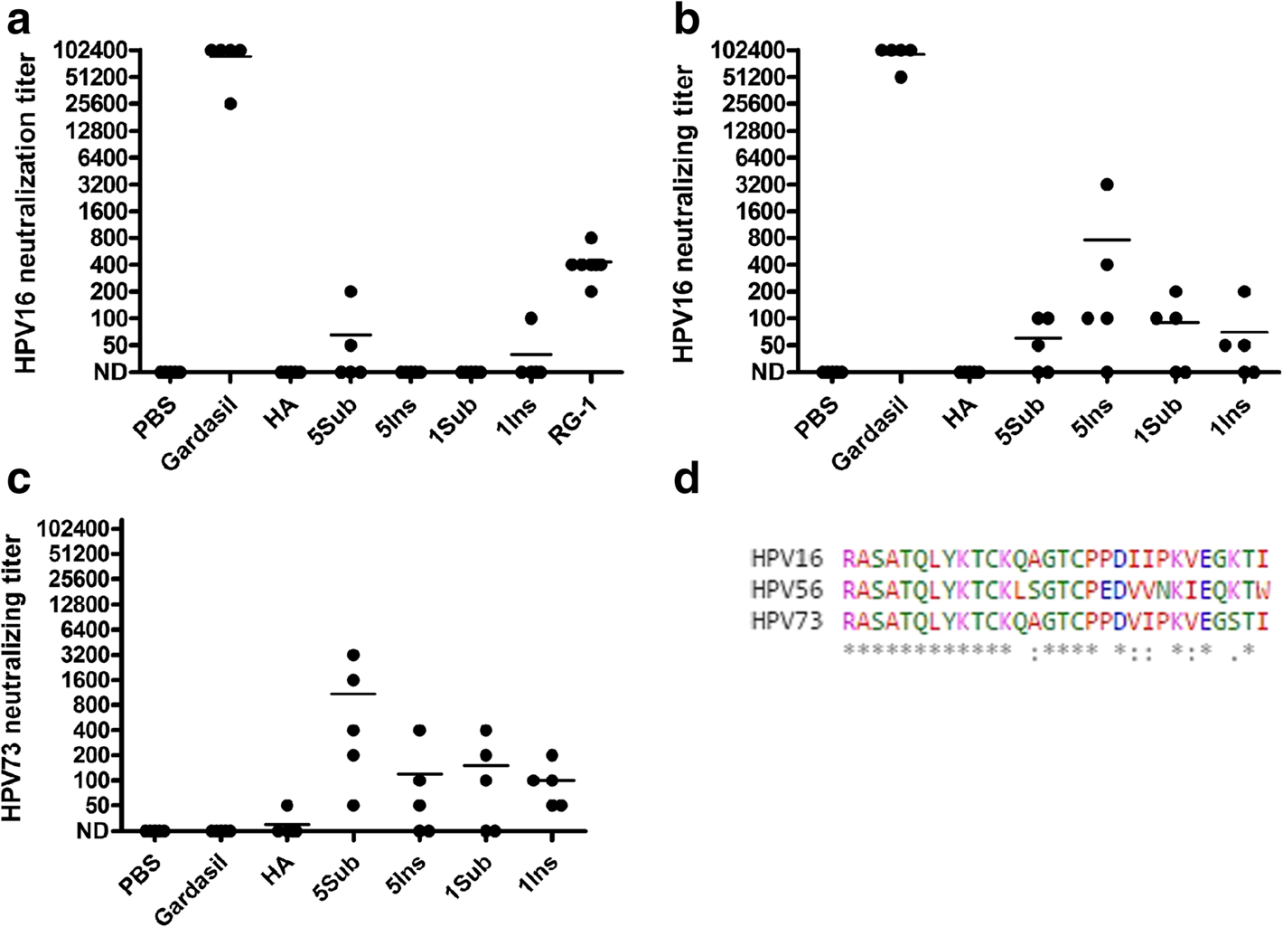
HPV16 and HPV73 neutralizing antibody elicited by vaccination of mice with L2-recombinant adenoviruses either with or without adjuvant. *Legend*: Balb/c mice (5/group) were vaccinated s.c. three times at 3 week intervals with 10^10^ L2 or HA-recombinant adenovirus particles either in the absence (**a**) or presence (**b**) of adjuvant (50 μg aluminum hydroxide gel and 5 μg monophosphoryl lipid A), or controls Gardasil (0.1x human dose) or phosphate-buffered saline (PBS). Serum was collected 3 weeks after the final immunization and two-fold dilutions tested by for *in vitro* neutralization of HPV16 (A, B) or HPV73 (**c**) pseudovirions. Purified RG-1 monoclonal antibody was included as a positive control for L2-specific neutralization and diluted from 1 μg/μL. A neutralization titer of <1:50 is considered Not Detectable (ND) and mean titer is shown. (**d**) A web-based T-coffee tool was used for the CLUSTAL W (1.83) multiple sequence alignment (http://www.ebi.ac.uk/Tools/msa/tcoffee/) comparison of the amino acid sequences of HPV16 L2 12–41 and the comparable regions of HPV56 and HPV73 color coded by amino acid side chain. The alignment is shown and identity is marked below with an asterisk and a conservative change with a colon

Sera collected 3 weeks after the third recombinant adenovirus immunization were titrated against HPV16 pseudovirions in an *in vitro* neutralization assay (Fig. 2a, b). All mice in the control (Gardasil) group produced high titer neutralizing antibodies. In the negative control (PBS and HA recombinant) groups none of the mice produced neutralizing antibodies, as expected. There was little evidence of HPV16 neutralizing antibody, as measured using the standard pseudovirion-based assay, in the sera of mice vaccinated with the L2 recombinant adenoviruses three times in the absence of adjuvant, despite reactivity in L2 ELISA. In the mice that received adjuvant each of the four recombinant adenoviruses induced low HPV16 neutralizing antibody titers (Fig. 2b). Similar results were obtained for cross-neutralization of HPV73 (Fig. 2c), a high risk member of the α11 species that is not targeted by the Gardasil 9 vaccine [9]. This likely reflects the near perfect conservation of the 17–36 epitope recognized by the cross-neutralizing monoclonal antibody RG1 [20], as shown in Fig. 2d.

### Immunization with L2-recombinant adenovirus protects mice against homologous but not heterologous HPV type challenge

**Fig. 3:**
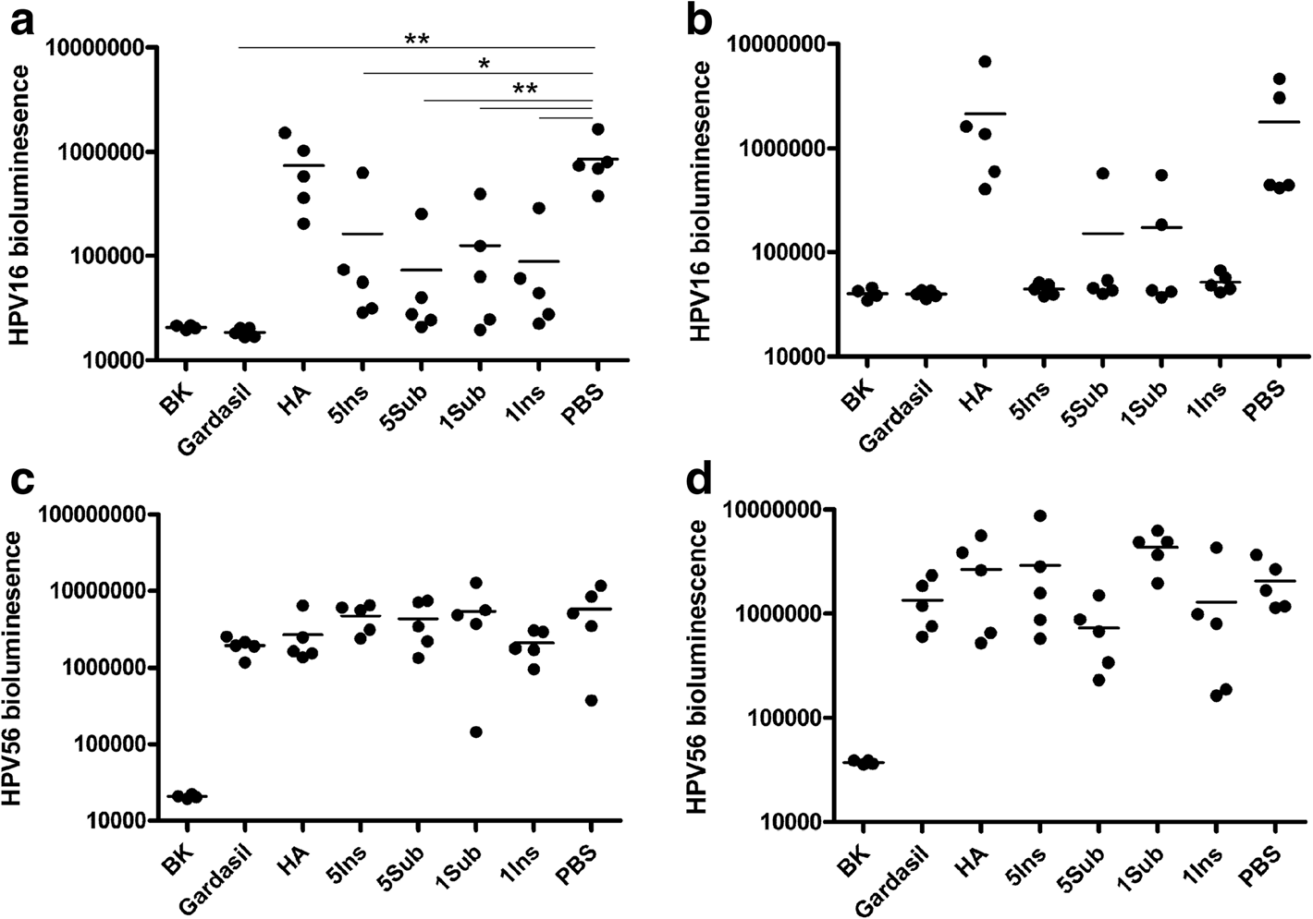
Protection of against vaginal challenge with HPV after vaccination with L2-recombinant adenoviruses either with or without adjuvant. *Legend*: Balb/c mice (5/group) were vaccinated s.c. three times at 3 week intervals with 10^10^ L2- or HA-recombinant adenovirus particles either in the absence (**a**,**c**) or presence (**b**,**d**) of adjuvant (50 μg aluminum hydroxide gel and 5 μg monophosphoryl lipid A), or controls Gardasil (0.1x human dose) or phosphate-buffered saline (PBS). Mice were challenged intravaginally with either HPV16 (**a**,**b**) or HPV56 (**c**,**d**) pseudovirions at 4 weeks after the final immunization. Three days later the infection was assessed by imaging of bioluminescence (RLU). The background bioluminescence of unchallenged mice (BK) is shown. **p < 0.01, *p < 0.05

**Fig. 4:**
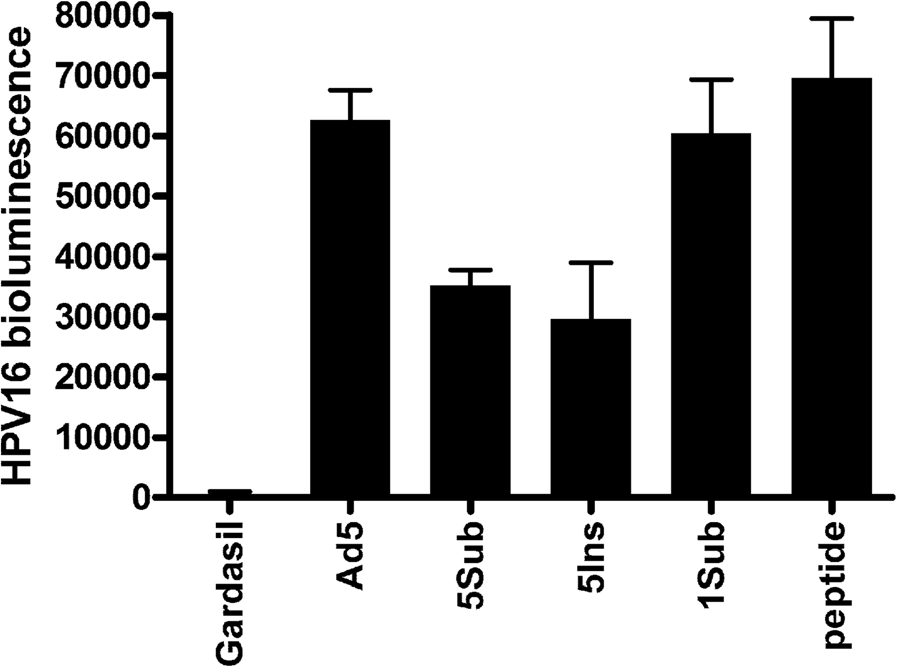
Partial protection against cutaneous challenge with HPV16 after vaccination with L2-recombinant adenoviruses without adjuvant. *Legend*: Balb/c mice (5/group) were vaccinated three times s.c. at 3 week intervals with either wild type or L2-recombinant Ad5 particles in the absence of adjuvant, or controls Gardasil (0.1x human dose) or synthetic HPV16 L2 17–36 peptide (20 nmol QLYKTCKQAGTCPPDIIPKV). Mice were shaved and then challenged on their belly with HPV16 pseudovirions after the final immunization and three days later the infection was assessed by imaging of bioluminescence (RLU). The data are presented as mean + SEM after subtraction of the mean background bioluminescence of unchallenged mice

The standard HPV in vitro neutralization assay protocol is poorly sensitive for L2-specific antibodies, whereas challenge models are both more biologically relevant and sensitive, i.e. L2-specific antibodies have a more potent effect in vivo. Therefore, vaccine efficacy was evaluated by challenging immunized mice with HPV pseudovirus (PsV) encapsidating a luciferase reporter. Immunized mice were first challenged intra-vaginally with HPV16 pseudovirus (Fig. 3a and b). The mice vaccinated with Gardasil 4, which contains HPV16 L1 VLP, were completely protected from experimental challenge (p < 0.01). The mice vaccinated in the absence of adjuvant with each L2-recombinant adenovirus were only partially protected from HPV16 challenge, but each was significant compared to mice immunized with PBS or the HA recombinant (p < 0.01 for 5Sub, 1Sub and 1Ins, p < 0.05 for 5Ins versus PBS). Cutaneous challenge was then conducted with HPV16 in a separate study of mice immunized using either HPV16 L2 17–36 synthetic peptide or the recombinant adenoviruses 5Sub, 5Ins or 1Sub without adjuvant. Partial protection was observed in those vaccinated with L2-recombinant adenovirus 5Sub and 5Ins, but not 1Sub or the HA recombinant (Fig. 4). Gardasil 4 was completely protective against HPV16 cutaneous challenge, whereas vaccination with HPV16 L2 17–36 synthetic peptide was not. The failure of the peptide alone to protect reflected its previously described lack of immunogenicity without T-cell help.

Vaccination with each L2 recombinant in the presence of adjuvant induced dramatically better protection in the vaginal challenge model. In a majority of recombinant-vaccinated mice, luminescence was reduced to a level indistinguishable from that of Gardasil 4-vaccinated animals, and every mouse immunized with recombinant adenoviruses 5Ins and 1Ins was fully protected (p < 0.05 for all four L2 recombinant adenoviruses). The HA recombinant control had no significant impact on HPV16 infection.

HPV16 and HPV56 are high risk viruses of the α9 and α6 species respectively, and the latter is not targeted by Gardasil 9. Since L2-specific antibodies are typically cross-protective, and we observed cross-species neutralization of HPV73 (α11 species), we examined protection against a divergent HPV type from a different species by heterologous intravaginal challenge with HPV56 pseudovirus (Fig. 3c and d), that diverges from the 30 amino acid HPV16 L2 epitope sequence at 9 positions (Fig. 2d). The PBS injected mice showed high luciferase activity, indicating they were infected. The Gardasil vaccinated group was not significantly protected against HPV56, consistent with the type-restricted response to L1 VLP and the absence of this type from the vaccine. Similarly, mice immunized with the L2-recombinant adenoviruses either with or without adjuvant also showed no significant protection against HPV56 irrespective of the recombinant virus construct used (Fig. 3).

## Discussion

Prior animal experiments by other investigators have demonstrated the ability to induce cross-protective antibodies against bovine and rabbit papillomaviruses by immunization with polypeptides derived from the N-terminus of L2 [35, 36]. L1 constitutes the bulk of the capsid structure and is immunologically dominant [37], and a minimal or undetectable L2-specific antibody response is observed in the setting of combined experimental L1 and L2 VLP vaccination or natural infection. Presumably the papillomaviruses have evolved to render these broadly cross-type protective L2 epitopes very poorly immunogenic to permit infection of a single host with multiple types. This might be achieved by holding L2 cross-type protective epitopes (whose sequences are conserved because of critical function during the infectious process) below the capsid surface until initiation of infection or simply by virtue of distant spacing of L2 epitopes on the capsid surface to prevent a typical L1 VLP-like response. High density virus-like display of conserved but sub-dominant protective L2 epitopes represents a promising strategy to induce potent and durable L2-specific antibody responses [24, 38]. Animal studies have demonstrated the feasibility of protective L2 epitope display in the Tobacco Mosaic Virus (TMV) with successful production of low titers (50–500) of protective antibodies when using RIBI adjuvant. When vaccinating with TMV displaying a conserved CRPV L2 epitope, robust protection was observed against CRPV but not ROPV, suggesting that cross-protection was much weaker than efficacy against homologous type challenge [39]. This result is quite consistent with the observations herein, including the moderate L2-specific antibody response and the type-restricted protection despite use of a relatively conserved protective epitope.

Adenoviruses are large non-enveloped DNA viruses which cause mostly short, transient upper respiratory and gastrointestinal infections in humans. In contrast to TMV, however, adenovirus can replicate in humans, and adenoviruses typically induce robust host immune responses. For decades now, the US military has been using live, orally administered single dose vaccines to immunize recruits against adenoviruses 4 and 7, with no reported significant adverse events [30, 31]. The major adenovirus capsid protein, hexon, is a 960 amino acid polypeptide present in 720 copies per viral particle. Hexon is capable of inducing a strong host antigenic response. Hexon contains 9 HVRs that possess epitopes which are responsible for antigenic diversity among adenovirus serotypes [25]. The amino acid sequences of HVRs can be modified with little effect on viability, and several investigators have used adenoviruses as viral vectors for capsid display of antigens to induce protective immunity against a host of human pathogens including pseudomonas, trypanosoma, plasmodium, and mycobacterium [27–29, 40–42]. The results obtained in this study demonstrate the feasibility of the capsid display of an L2 epitope by insertion into HVR1 and 5 of the adenovirus 5 hexon.

Experiments using influenza epitopes [43] have shown that HVR length can affect the immune response probably due to the position and conformation of the HVRs within the hexon sequence. Specifically, HVR5 was shown to be more permissive for larger sequence insertions compared to HVR2, and this correlated with higher titer immune responses. Due to the limited capacity of the adenovirus HVRs, it was not clear whether insertion or substitution of the HVR with the first ~100 residues of L2 would be feasible without compromising replication competence. However, both our fairly short HPV L2 substitutions and insertions were successfully incorporated in the hexon structure without compromising virion replication. Further, both insertion and substitution recombinant adenoviruses elicited similar L2 antibody responses.

We have also demonstrated that the recombinant vaccine constructs were capable of inducing neutralizing antibodies in sufficient titers to confer protection against HPV16, as evidenced by the challenge experiments. Use of an adjuvant (alum + monophosphoryl lipid A (MPL), similar to that incorporated in the licensed HPV vaccine Cervarix) enhanced immunogenicity. Given that the recombinant adenoviruses are non-replicating in mice, we postulate that the adjuvant was therefore relevant to boost antibody response. It is reasonable to expect that in a human, our viable recombinant viruses might be independent of co-administered adjuvant given the likelihood that viral lysis of infected cells will trigger local inflammation [44], and that the infection is likely to persist for days to a few weeks, as do infections with the military vaccine strains [45].

The L2-recombinant adenoviruses failed to induce cross-protective immunity against HPV56. This is surprising in light of the promising results obtained when vaccinating with KLH-coupled HPV16 L2 17–36 peptide in Freund’s adjuvant [20, 46] or when this epitope is inserted into the DE loop of the HPV16 L1 VLP capsid [47, 48]. Several explanations are proffered. First, the L2 epitopes of HPV16 and HPV56 may not be sufficiently similar to provide cross-protection given the weak HPV16 L2-specific response measured by ELISA and neutralization assays. This is the most likely reason as there is considerable difference, 9 of 30 residues, between these types in the L2 28–41 neutralizing epitope region [21, 22], as can be seen in Fig. 2d, and the ability to cross-neutralize HPV73 which is more similar in this region (only 2 changes in 30 residues, one of which is conservative). Second, the HPV L2 epitope as displayed in our recombinant adenoviruses, may suffer from non-native spatial conformation that could affect the breadth of the immune response or promote the production of non-neutralizing antibodies. Recent work has shown that cross-protection against the HPV L2 epitope is indeed affected by the flexibility of the epitope during capsid display [49], i.e. greater cross-protection was observed when one end of the L2 epitope was free. Third, in natural infections, cleavage of furin near the N terminus of L2 causes one end of the epitope used here to be free, presumably affecting conformation of the L2 epitope. The capsid display recombinant adenoviruses lacked furin cleavage sites, and were inserted or substituted into the hexon with both ends attached. It is reasonable to try to increase flexibility in the L2 display by using glycine-serine-glycine (GSG) flankers for example, or by inserting furin cleavage sites to help mimic native configuration of L2 in a replication-capable setting. Insertion/substitution of L2 into the other 7 HVRs might increase efficacy, and it may be advantageous to insert L2 into multiple HVR in a single construct to increase epitope density, which is associated with higher immunogenicity [38]. An alternative approach to enhance the breadth of protection is to insert L2 epitopes from different HPV types into different HVR, as described by Nieto et al. for AAV [50], or to insert a concatemer of L2 epitopes of different HPV types into a single HVR [51]. Alternatively, the use of a consensus sequence might be considered but this could reduce responses to key types such as HPV16.

Prior adenovirus exposures are not uncommon in human adults and there is a general concern about the potential impact of pre-existing immunity against a recombinant adenovirus based vaccine in (future) clinical application. A study using an Ad5 based tuberculosis vaccine did not confirm a postulated deleterious effect of pre-existing antibodies on the antigenicity of the vaccine constructs that were tested [52]. However, this vaccine is replication deficient and pre-existing antibodies may be even more problematic for live vaccines. Strategies to bypass this potential problem include the use of less-commonly occurring human adenovirus types.

## Conclusions

Our studies demonstrate the antigenicity of capsid display of the HPV L2 epitope using an adenovirus vector. This represents an initial step in the search for an alternative to the L1 VLP based prophylactic vaccines in use today. Continued optimization of the display of L2 by hexon clearly will be required. If successful and safe, L2-recombinant adenovirus vaccines could potentially be produced inexpensively, and simply administered in a heat-stable single oral dose for improved patient access and compliance.

## Methods

### Insertion of L2 epitope into HVRs

Constructs containing the L2 sequence either as a substitution or insertion into adenovirus 5 hexon protein hypervariable (HVR) regions 5 or as a substitution into HVR1 were generated by overlapping PCR (Table 1) using primer sets shown below 5′ to 3′.

For construct 1Sub,

Primer 1 cgggtgggcaggtgccggcctgcttgcaggtcttgtacagctgggtggcgctggctctAGCTTCATCCCATTCGCAAGG

and Primer 2 gcaggccggcacctgcccacccgatatcatccccaaggtggagggcaagaccatcGTATTTGGGCAGGCGCCTTATTCTGG

For construct 5Ins,

Primer 1 cgggtgggcaggtgccggcctgcttgcaggtcttgtacagctgggtggcgctggctctCTCAGTAGTTGAGAAAAATTGC

and Primer 2 gcaggccggcacctgcccacccgatatcatccccaaggtggagggcaagaccatcGCGACCGCAGGCAATGGT

For construct 5Sub, Primer 1

5' cgggtgggcaggtgccggcctgcttgcaggtcttgtacagctgggtggcgctggctctCTCAGTAGTTGAGAAA AATTGC

and Primer 2 gcaggccggcacctgcccacccgatatcatccccaaggtggagggcaagaccatcACTCCTAAAGTGGTATTGTAC

For all three reactions, CGGCGTGCTGGACAGGGGCCC and GCTGGCTCCGTCAACCC were used as outside primers. The PCR products were cloned into plasmid pCR2.1 using the TopoTA system (Invitrogen) and were subcloned into shuttle plasmid pJMG (Ad5 nucleotides 13255 – 21562, including all of the hexon gene).

For construct 1Ins, a DNA sequence was directly synthesized (Biobasic, Inc., Ontario, Canada; GGGCCCTACTTTTAAGCCCTACTCTGGCACTGCCTACAACGCCCTGGCTCCCAAGGGTGCCCCAAATCCTTGCGAATGGGATGAAGCTGCTAGAGCCAGCGCCACCCAGCTCTACAAGACCTGCAAGCAGGCCGGCACCTGCCCACCCGATATCATCCCCAAGGTGGAGGGCAAGACCATCACTGCTCTTGAAATAAACCTAGAAGAAGAGGACGATGACAACGAAGACGAAGTAGACGAGCAAGCTGAGCAGCAAAAAACTCACGTATTTGGGCAGGCGCCTTATTCTGGTATAAATATTACAAAGGAGGGTATTCAAATAGGTGTCGAAGGTCAAACACCTAAATATGCCGATAAAACATTTCAACCTGAACCTCAAATAGGAGAATCTCAGTGGTACGAAACTGAAATTAATCATGCAGCTGGGAGAGTCCTTAAAAAGACTACCCCAATGAAACCATGTTACGGTTCATATGCAAAACCCACAAATGAAAATGGAGGGCAAGGCATTCTTGTAAAGCAACAAAATGGAAAGCTAGAAAGTCAAGTGGAAATGCAATTTTTCTCAACTACTGAGGCGACCGCAGGCAATGGTGATAACTTGACTCCTAAAGTGGTATTGTACA). This includes an *EcoRV* site to facilitate screening of recombinant adenoviruses, and encodes Ad5 hexon (19160–19703) with an insertion of HPV16 L2 12 to 41 (RASATQLYKTCKQAG TCPPDIIPKVEGKTI) into HVR1. This entire sequence was directionally inserted into pJMG using the flanking *ApaI* and *BsrGI* sites.

### Generation, isolation and purification of recombinant adenovirus

For 1Sub, 5Sub and 5Ins recombinant adenoviruses, engineered hexon DNA fragments were introduced into the full-length Ad5 genomic plasmid CP08 by phage lambda *red*-mediated recombination in *E. coli* between CP08 and the shuttle plasmids described above [53], CP08 carries a full-length Ad5 genome with an insertion of *lacZ* in the hexon gene [29]. Following recombination, *E. coli* strains that contained the desired recombinants were identified by restriction enzyme digestion of miniprep DNAs with *EcoRV*. To generate virus from recombinant plasmids, linear viral genomic DNA liberated from the plasmid by digestion with *PacI* was introduced into 293 cells by calcium phosphate precipitation [54]. Individual plaques were picked and amplified in 293 cells. Lysates were screened by immunoblotting for reactivity with the L2 antibody RG-1 to confirm that they contained the L2 peptide. Recombinant adenovirus 1Ins was prepared by recombination in transfected 293 cells between the shuttle plasmid and virion DNA derived from a viable Ad5 recombinant bearing a *Plasmodium falciparum* circumsporozoite epitope (CSP) in hexon [55]. The recipient DNA was digested with *NdeI*, which cleaves the recombinant at a single site in hexon, reducing plaque formation by the parental virus. Plaque purified recombinant viruses were amplified in 293 cells and were screened for RG-1 reactivity to detect the L2 epitope and with a CSP-specific monoclonal antibody to confirm loss of the allelic malaria epitope. All recombinant adenoviruses were plaque-purified and virus particles were prepared by CsCl density gradient centrifugation and quantified as described previously [56]. A control recombinant that displays an epitope derived from influenza virus A hemagglitinin (HA) was prepared similarly (C. Deal and G. Ketner, unpublished).

### Immunization of mice and HPV challenge

All animal experiments were performed with the prior approval of the Johns Hopkins University Animal Care and Use Committee. Female 3–6 week Balb/c mice were obtained from the National Cancer Institute and were immunized by subcutaneous injection with 1 × 10^10^ recombinant virus particles suspended in PBS (non-adjuvant group) or mixed with 50 μg aluminum hydroxide gel and 5 μg monophosphoryl lipid A in a total volume of 150 μL/dose, or as a control synthetic HPV16 L2 17–36 peptide (20 nmol QLYKTCKQAGTCPPDIIPKV). Mice were boosted with the same recombinant virus dose at 3 weeks and 6 weeks after initial vaccination. Serum was collected every 3 weeks. Four weeks after the third immunization, mice were treated with progesterone (Depo-Provera) [57]. Four days after treatment, HPV pseudovirus (PsV) encapsidating a luciferase reporter was used for vaginal challenge, before and after induction of local epithelial trauma using an endocervical brush. Each mouse was challenged with HPV pseudovirions in 20 μL (estimated at 10^9^ particles/mouse based on L1 content). An equal volume of 3 % CMC (Carboxymethylcellulose sodium salt, C5013 Sigma, St Louis MO, USA) in PBS was added to make a total virus challenge volume of 40 μL per mouse. Mice were anesthetized with isoflurane inhalation to effect prior to administration of virus. Half of the challenge dose (20 μL) was injected into the mouse vaginal vault, followed by insertion of a cytobrush cell collector that was turned both clockwise and counter-clockwise 15 times to induce trauma. After removal of the cytobrush, the remaining half of the inoculum was deposited in the vagina. Three days later, the vaginal tracts were instilled with luciferin (20 μL at 7 mg/mL) and the genital area of anaesthetized mice was imaged for 10 min with a Xenogen IVIS200 system [51, 58]. For skin challenges, a patch on the belly of each anesthetized Balb/c mouse was closely shaved, first with an electric razor (Oster Golden 45, blade 40), then repeatedly with a scalpel blade to remove all traces of hair, and challenged by application to the shaved skin of 3 × 10^9^ HPV pseudovirions in 10 μL of 0.6 % CMC. Three days later, the mice were anesthetized and injected with luciferin (100 μL at 7 mg/ml), and their images were acquired for 10 min with a Xenogen IVIS 200. Equally sized areas encompassing the site of inoculation were analyzed using Living Image 2.20 software, and plotted after background subtraction. Using Graphpad software, the results of the challenge were recorded [59, 60].

### ELISA

For adenovirus, 96 well plates (Immunolon) were coated overnight at RT with 7.85 × 10^11^ wild type adenovirus particles/plate in PBS. For L2, 96 well NUNC Maxisorp plates were coated and incubated overnight at 4 °C with a bacterially-expressed L2 antigen comprising residues 11–88 of HPV16 and seven other alpha HPVs in tandem (100 ng/well of α11-88x8, an antigen described previously [61, 62]). The coated plates were then washed three times with PBST (0.01 % Tween 20) and three times with PBS. The wells were then blocked with 1 % BSA in PBS for 1 hour at room temperature. Mouse serum was serially diluted in 1 % BSA in PBS, added to the wells and incubated at RT for 1 hour. Plates were washed again as previously described with PBST and PBS. HRP-conjugated sheep anti-mouse antibody (GE Healthcare) was added at a dilution of 1:5000 in 1 % BSA in PBS and incubated at RT for 1 hour. Plates were washed one final time with PBST and PBS. The plates were developed for 15 (adenovirus) or 30 (L2) minutes with the ABTS Peroxidase Substrate System (KPL) and then optical density was measured at 405 nm using a microplate reader. Experiments were performed in triplicate and each experiment had duplicates of each sample. The endpoint ELISA titer was determined as the highest dilution at which the average optical density of the sample was greater than twice the average of the negative control.

### Recombinant adenovirus neutralization

1 ml of medium containing 200 pfu of the adenovirus-HPV recombinant was mixed with an equal volume of serially diluted mouse sera. The mixture was incubated at 37 °C for 30 minutes and then 1 mL added to each of two 6 cm cell culture dishes containing a 293 cell monolayer. The virus was adsorbed for 2 hours and the plates were overlaid with agar medium (MEM without phenol red containing 0.9 % Bacto-Agar, 2 % FBS, penicillin and streptomycin). On the third and sixth day later, the cells were fed with 2.5 mL of agar medium. On day six, the agar medium contained neutral red and plaques were counted daily until their numbers became constant [56, 63].

### HPV neutralization assays

HPV 16 pseudovirus encapsidating a secreted alkaline phosphatase (SEAP) reporter plasmid was incubated with mouse sera for 1 h at RT [64]. The mixture was used to infect cells in a 96-well plate. 72 h post-infection, the supernatant was collected and SEAP activity was measured using a highly sensitive chemiluminescent reporter system. Serum neutralization titers were defined as the highest dilution that caused at least a 50 % reduction in SEAP activity [33].

### Statistical analysis

Data were imported into GraphPad Prism 4.03 for preparation of box plots, bar charts or statistical analysis (mean + SEM). Comparisons of protection between groups were made by one way ANOVA with Bonferroni correction. Data are presented as mean + SEM.
